# Secondary correction of nasal deformities in cleft lip and palate patients: surgical technique and outcome evaluation

**DOI:** 10.1186/s13005-016-0132-y

**Published:** 2016-12-01

**Authors:** Gabor Vass, Gabor Mohos, Zsofia Bere, Laszlo Ivan, Janos Varga, Jozsef Piffko, Laszlo Rovo

**Affiliations:** 1 Department of Dermatology and Allergology, University of Szeged, Korányi fasor 6, 6720 Szeged, Hungary; 2 Department of Oral and Maxillofacial Surgery, University of Szeged, Kálvária sgt. 57, 6725 Szeged, Hungary; 3 Department of Oto-Rhino-Laryngology and Head and Neck Surgery, University of Szeged, Tisza L. krt. 111, 6725 Szeged, Hungary

**Keywords:** Cleft lip and palate, Secondary rhinoplasty, Standard surgical technique, Rhinoplasty outcome evaluation questionnaire

## Abstract

**Background:**

Nasal deformity associated with cleft lip and palate is a highly challenging reconstructive problem in rhinoplasty. In the literature, several operative solutions and evaluation methods have been described, however these do not offer a standard procedure for the surgeon. Our aim was to standardize our surgical technique—as much as the uniqueness of each case allowed it—based on the most frequent deformities we had faced; and to evaluate our results via a postoperative patient satisfaction questionnaire.

Between 2012 and 2014 12 consecutive patients with combined cleft lip and palate deformities underwent secondary nasal and septal correction surgery with the same method by the same surgeon. The indications of surgery were, on one hand, difficult nasal breathing and altered nasal function (tendency for chronic rhinosinusitis) and on the other hand the aesthetic look of the nose. No exclusion criteria were stated. In our follow-up study we evaluated our results by using a modified Rhinoplasty Outcome Evaluation (ROE) questionnaire: patients answered the same four questions pre- and postoperatively. Data were statistically analyzed by *t*-test.

**Results:**

Based on the questionnaire, all patients experienced improvement of nasal breathing function, improved appearance of the nose and less stigmatization from the society. According to the *t*-test, all scores of the four questions improved significantly in the postoperative 4–6 months, compared with the preoperative scores.

**Conclusions:**

In our opinion with our standardized surgical steps satisfactory aesthetic and functional results can be achieved. We think the modified ROE questionnaire is an adequate and simple method for the evaluation of our surgical results.

## Background

Cleft lip and palate (CLP) deformities are among the most common congenital malformations. The overall incidence of cleft palate with or without cleft lip is 1 case in approximately 1000 live births in the USA and in Europe [1, 2]. In Hungary the incidence of combined oro-facial clefts is 2 in 1000 live births [2]. Although CLP together occur more commonly in males, isolated cleft palate is more common in females [1, 2].

Surgical correction of CLP should be performed before the first year of age, usually between 3 and 6 months-of-age, prior to speech development. The aim of the operation is to reunite all tissue layers of the lip, to reposition the nasal septum and to separate the oral and nasal cavities; and restore the valve function of the soft palate [1, 2].

If this adequate primary surgical correction of CLP fails, the consequentially developing nasal deformity associated with CLP is one of the most challenging reconstructive problems in rhinoplasty. The characteristic cleft lip nose represents a stigma for the patient. This results from a combination of altered anatomy, surgical scaring from previous reconstructive operations and includes deformities of the septum, nasal pyramid, malformation of the nasal tip and malposition of alar cartilages.

The indication for surgery is on one hand the difficult nasal breathing and altered nasal function (tendency for chronic rhinosinusitis) and on the other hand the aesthetic look of the nose, both of which may affect the patient’s quality of life negatively and can cause heavy psycho-social burden for them. Accompanying nasal deformities are mainly characterized by a shortened columella, a depressed nasal tip, bilateral dislocation of the alar cartilage, eversion of the alar bases and nasal obstruction [3–6].

Although numerous secondary rhinoplasty methods have been described in the literature for lengthening of the columella, or for grafting techniques, no standardized technique exists. Our aim with this study was to somehow standardize the secondary rhinoplasty operations of patients with CLP at our University of Szeged, Albert Szent-Györgyi Medical Center—as much as the uniqueness of each case allowed it—based on the most frequent deformities we had observed. In order to evaluate our surgical results, we designed a follow-up study to compare the pre- and postoperative functional and aesthetic results with an adopted Rhinoplasty Outcome Evaluation (ROE) questionnaire.

## Methods

Between 2012 and 2014 12 consecutive patients with combined CLP deformities underwent nasal reconstructive surgery performed by the same operative team in cooperation with other departments of our University. Every patient already underwent dental and maxillo-facial rehabilitation (orthodontia, oronasal fistula closure, bimaxillary orthognathic surgery, etc.), no further surgical intervention was planned in connection with their congenital malformation. Ten patients had unilateral and two patients had bilateral cleft lip deformity. They included four males and eight females, their ages varied from 17 to 26 with a mean age of 21 years.

There were no exclusion criteria and only two inclusion criteria were set: patients had to have CLP and had to be older than 16. All patients signed the informed consent documents of the operation. As all surgical methods have already been published in the literature; our innovation was to combine of the different techniques into a standard surgical protocol, thus no ethical approval was necessary.

After analyzing the pathological anatomy of the nose the following surgical steps were used generally: philtrum surgery, septal surgery, alar and nasal tip surgery and nasal pyramid reposition.

Surgery was always carried out under general anesthesia via an open rhinoplasty approach. The columellar skin was in each case lengthened via a V-Y plasty of the philtrum area. During the septal surgery part an interalar approach was used, followed by subperichondral and subperiostal tunneling. The deviated cartilagineous and bony parts were resected, the remaining septal plates were then positioned back to the midline and, if available, septal cartilage was harvested for grafting. If any severe deviation of the septal dorsum was visible, dorsal grafts were used unilaterally or bilaterally on one hand to straighten it, on the other hand to adjust the height of the dorsum. The anterior septal base was then sutured to the anterior nasal spine, or if this was dislocated, to the midline [3, 6].

**Fig. 1:**
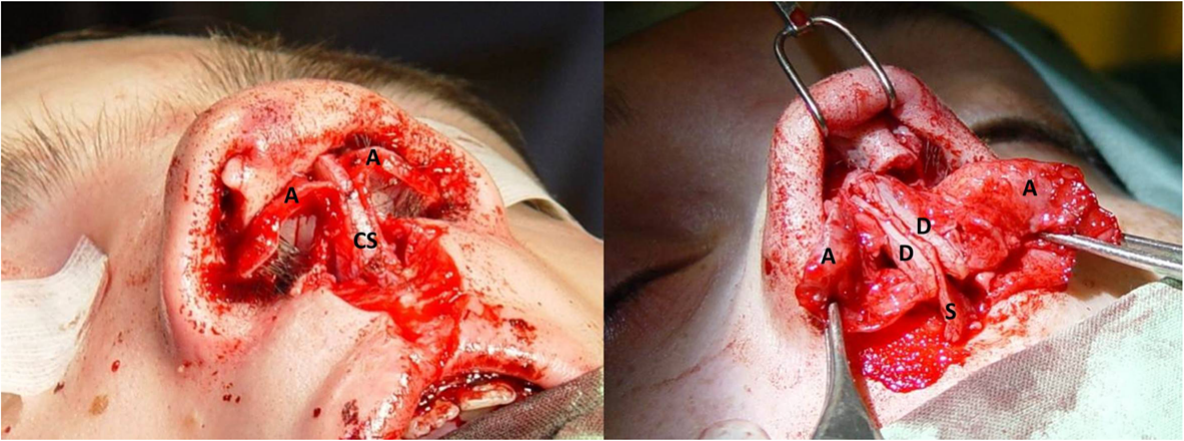
nasal grafting with septal cartilage; columella strut graft on the left and dorsal graft on the right picture (A: alar cartilage, CS: columella strut, D: dorsal graft, S: nasal septum)

Autologous nasal septum cartilage grafts and, if necessary, autologous cartilage from the concha, were used to rebuild the nasal framework in the second step. The lower lateral cartilage on the cleft side was positioned into a more medial and prominent position and the two medial crural cartilages were sutured together with the columella strut to set the tip projection. If the lateral crus was buckled, strengthening was done with an onlay conchal graft. Occasionally a shield graft was used to define the nasal tip (Fig. 1) [3, 4, 6].

Bony pyramid surgery, if rarely necessary, consisted of hump resection, medial and lateral osteotomies and repositioning of the nasal bone [3].

All 12 patients received both columella and dorsal grafts, harvested 11 times from the nasal septum and once from the ears; shield graft or tip graft was used in three patients fabricated from septal cartilage and an onlay alar graft, harvested from the concha, was necessary in one case.

To measure the patient satisfaction, we adapted the ROE questionnaire, which was first described by Alsarraf et al to measure facial aesthetic surgery outcome [7]. The questionnaire was modified by Arima et al for patients having rhinoplasty [8]. Our adapted ROE questionnaire asks the same four questions before and after surgery, the patient has to score each question on a scale between 0 to 4 points, where 0 represents the least satisfaction and 4 represents the highest one:

1. How much do you like the appearance of your nose?
2. How much can you breathe through your nose?
3. How much do you think your friends and those close to you like your nose?
4. Do you think the appearance of your nose limits your social or professional activities?

Scores for each individual question were compared using a *t*-test (IBM SPSS Statistics ver20), p was considered significant at 0.005.

## Results

**Fig. 2:**
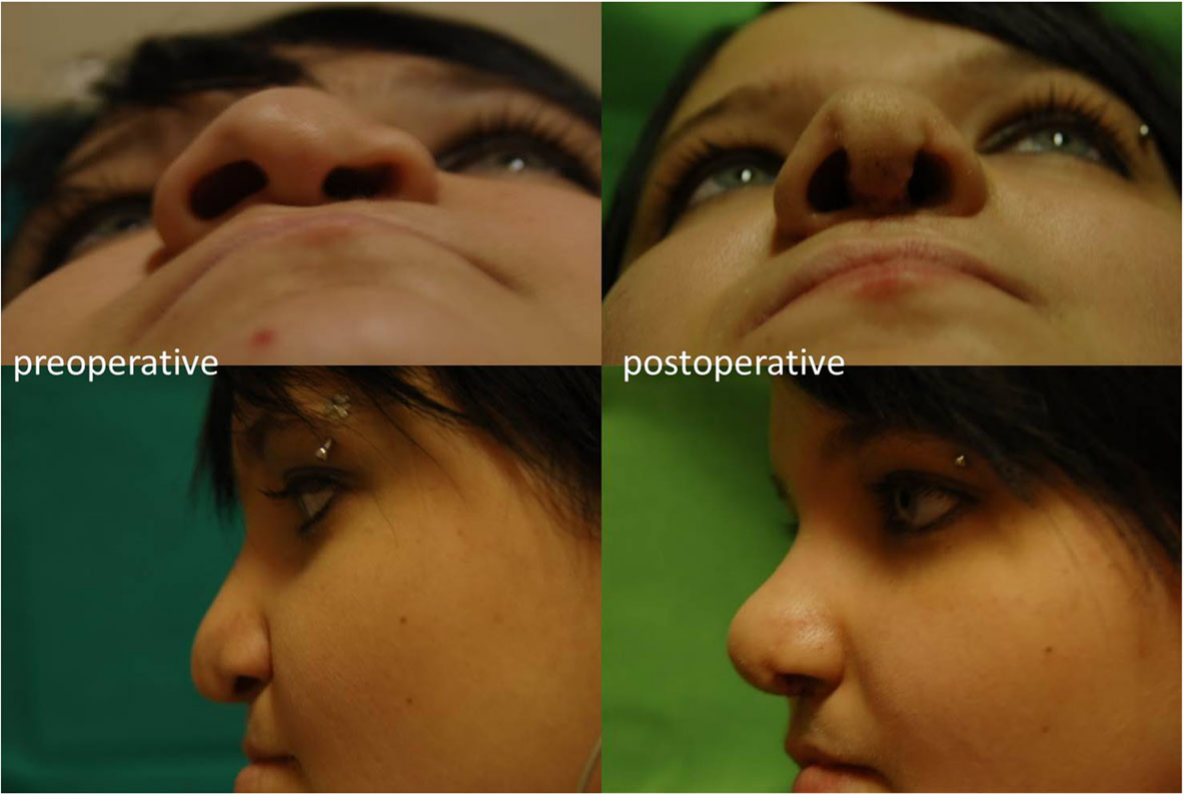
surgical results; lengthened columella, elevated nasal tip and set tip projection, adjusted dorsal height and symmetry given for the nostrils

**Fig. 3:**
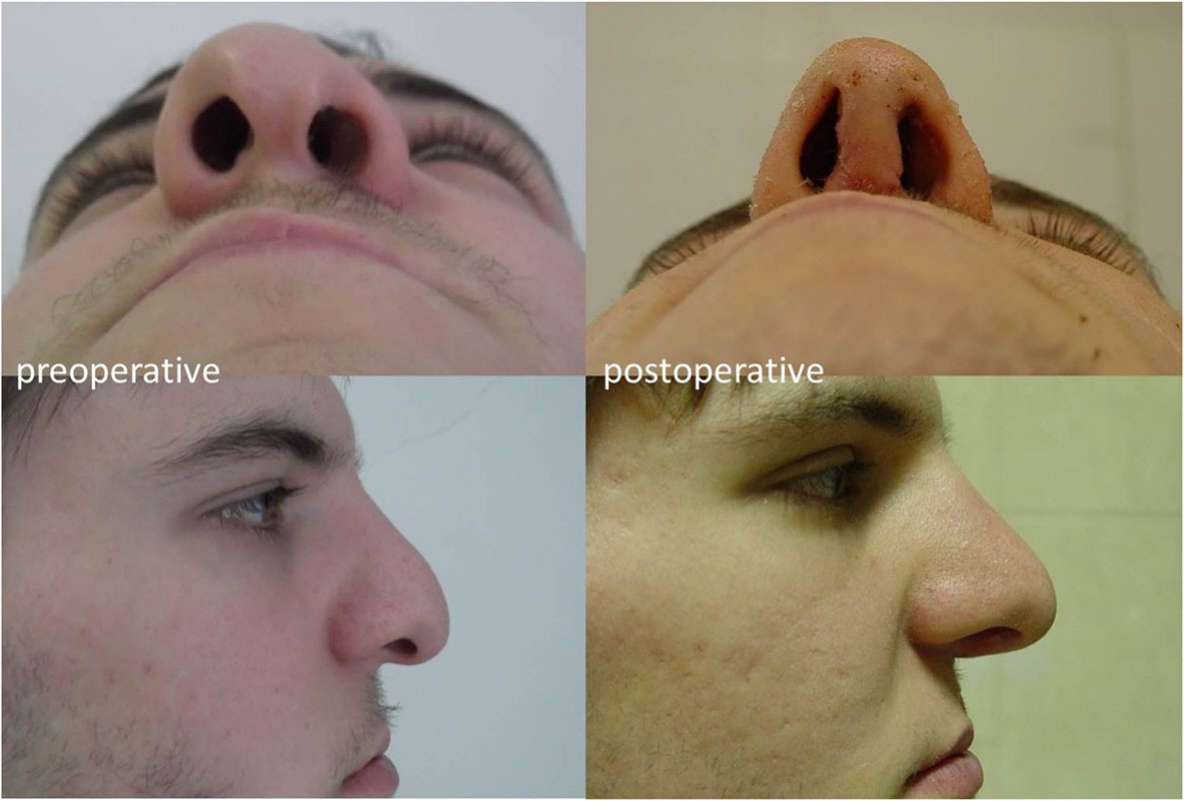
Surgical results; lengthened columella, elevated nasal tip and set tip projection and symmetry given for the nostrils

With the above detailed standardized surgical steps adequate aesthetic and functional results were achieved in all patients as shown in the results of the questionnaire and by the follow-up examinations of the patients (Figs. 2 and 3).

Table 1 shows the four questions of the ROE questionnaire and the statistical data.

**Table 1:** Results of the questionnaire

	preoperative		postoperative	
	mean ± SD	median	mean ± SD	median
How much do you like the appearance of your nose?	0.6 ± 0.6	1	3.5 ± 0.5	4
How much can you breathe through your nose?	2.1 ± 0.9	2	3.7 ± 0.5	4
How much do you think your friends and those close to you like your nose?	2.8 ± 0.8	3	3.8 ± 0.4	4
Do you think the appearance of your nose limits your social or professional activities?	2.8 ± 1.0	3	3.9 ± 0.3	4
Total Score	7.8 ± 0.8		15.0 ± 1.0	

The four questions asked are listed in the first column, patients had to score each question with 0–4 points, where 0 was the least and 4 was the highest value; the total score was 16 points. The other two columns show the answer scores of each question pre- and postoperatively, in detail the mean ± SD and the median (most frequently given score) values. The last row summarizes the total score of the questionnaire given by all 12 patients. There is a significant improvement between the pre- and postoperative mean values for each individual question (*p* = 0005).

**Fig. 4:**
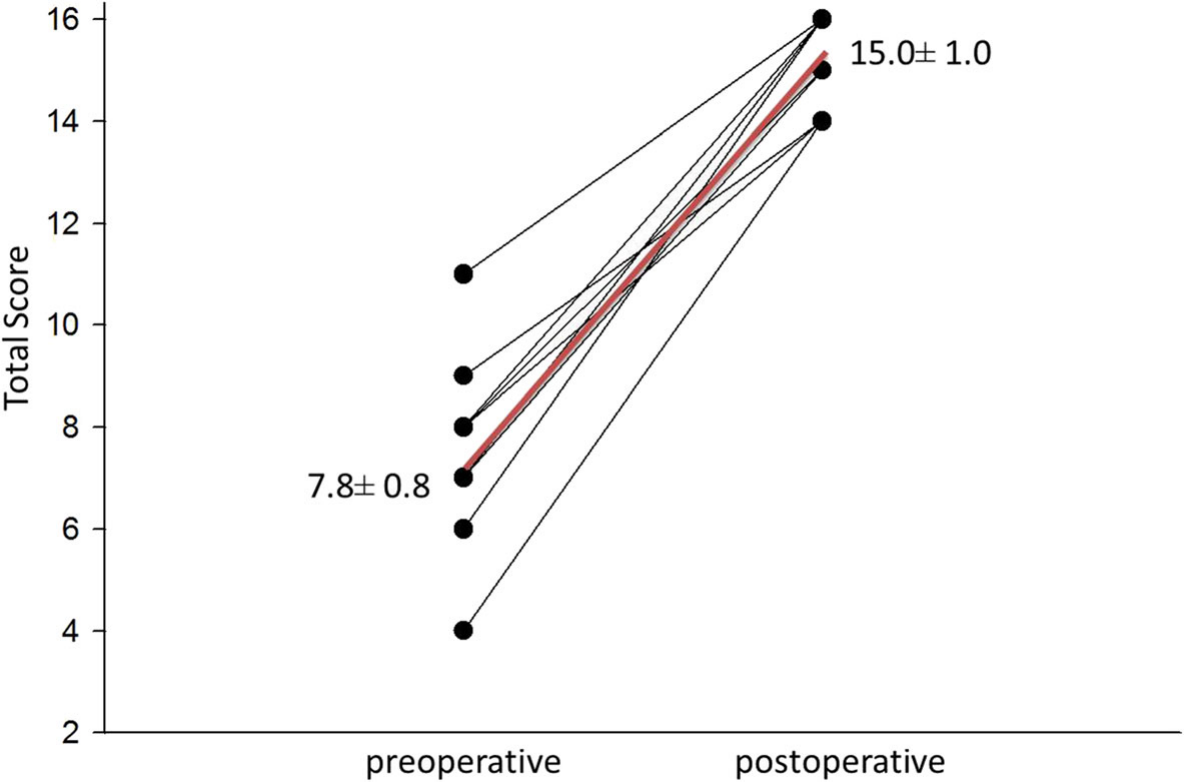
Pre- and postoperative changes in Total Score (total points given by each patient for all questions). Each dot represents the total given score of one patient for the all of the four questions, (less than 12 dots and lines result from overlapping scores, i.e. the same score was given by more than one patient for the same question; maximum points: 16). The red line shows the tendency of increase. Average mean ± SD is also presented

All patients were most satisfied with the postoperative appearance of their nose. The opinion of others about the appearance of the patient’s nose after surgery also improved. However, the least difference between the pre- and postoperative scores was with the last question, which could mean that the nasal deformity does not suppose an important limitation in Hungary for social and professional activities in these CLP patients (Fig. 4).

## Discussion

If the child receives the adequate functional surgery before the first year of age, usually there is no need for secondary rhinoplasty. In every other case secondary septo-rhinoplasty is advised optimally after the adolescence age but not before the age of 16 [5].

Unilateral or bilateral clefts can be distinguished generally. The difference between the nasal deformities associated with unilateral versus bilateral clefts and our surgical solution is presented in Table 2.

**Table 2:** Nasal deformities associated with unilateral and bilateral clefts and our surgical solutions

unilateral cleft	bilateral cleft	surgical solutions unilateral/bilateral
perpendicular plate deviates towards the cleft side	shortened columella	resection of the deviated bony septum/columellar skin gained by V-Y plasty
nasal spine deviates towards the non-cleft side	lack of septal cartilage in the anterior columellar region	not corrected/columella strut graft is used
bony pyramid deviates towards the non-cleft side	downward rotation of the nasal tip	bony pyramid replacement vis medial and lateral osteotomies/tip projection provided by the columella strut
lateral displacement of the alar base at the cleft side	bifidity of the nasal tip	lower lateral cartilage replacement/tip refinement with sutures and/or shield/tip grafting
downward displacement of the alar cartilage at the cleft side	buckling of the lateral crura on both sides	in both cases alar cartilage replacement and the two medial crural cartilages sutured together with the columella strut
asymmetry/bifidity of the dome area	usually no severe septal deviation	in both cases tip refinement with sutures and/or grafting
down position of the medial crus at the cleft side	downward rotation of the alar cartilage	in both cases tip projection provided by the columella strut

## Conclusions

In our opinion with the above mentioned operative protocol we were able to standardize our surgical technique in the secondary septo-rhinoplasty of patients with CLP. Skin incisions, cartilage harvesting and grafting, endonasal surgery and re-establishment of the nasal framework were successfully unified thus providing a more predictable functional and aesthetic outcome for the already psychosocially affected CLP patients.

Statistical comparative analysis of the pre- and postoperative data from our ROE questionnaire confirmed, that with our standardized surgical protocol improved aesthetic and functional results and good patient satisfaction rates were achieved.

We think our modified ROE questionnaire is an adequate and simple method for the evaluation of the surgical results of secondary septo-rhinoplasty among patients with CLP.

## Abbreviations

CLP: Cleft lip and palate
ROE: Rhinoplasty outcome evaluation
